# The Validity and Reliability of a New Intelligent Three-Dimensional Gait Analysis System in Healthy Subjects and Patients with Post-Stroke

**DOI:** 10.3390/s22239425

**Published:** 2022-12-02

**Authors:** Yingpeng Wang, Ran Tang, Hujun Wang, Xin Yu, Yingqi Li, Congxiao Wang, Luyi Wang, Shuyan Qie

**Affiliations:** 1 Department of Rehabilitation, Beijing Rehabilitation Hospital, Capital Medical University, Beijing 100144, China; 2 Beijing Rehabilitation Medical College, Capital Medical University, Beijing 100144, China

**Keywords:** gait analysis, Odonate, stroke, reliability, validity

## Abstract

Odonate is a new, intelligent three-dimensional gait analysis system based on binocular depth cameras and neural networks, but its accuracy has not been validated. Twenty-six healthy subjects and sixteen patients with post-stroke were recruited to investigate the validity and reliability of Odonate for gait analysis and examine its ability to discriminate abnormal gait patterns. The repeatability tests of different raters and different days showed great consistency. Compared with the results measured by Vicon, gait velocity, cadence, step length, cycle time, and sagittal hip and knee joint angles measured by Odonate showed high consistency, while the consistency of the gait phase division and the sagittal ankle joint angle was slightly lower. In addition, the stages with statistical differences between healthy subjects and patients during a gait cycle measured by the two systems were consistent. In conclusion, Odonate has excellent inter/intra-rater reliability, and has strong validity in measuring some spatiotemporal parameters and the sagittal joint angles, except the gait phase division and the ankle joint angle. Odonate is comparable to Vicon in its ability to identify abnormal gait patterns in patients with post-stroke. Therefore, Odonate has the potential to provide accessible and objective measurements for clinical gait assessment.

## 1. Introduction

Gait abnormalities are common in neurological and orthopedic disorders, and quantitative gait analysis has been widely used in clinical gait assessments to understand their biomechanical and pathophysiological mechanisms and predict treatment outcomes [1,2,3,4]. Stroke is one of the main causes of disability and mortality in elderly people and can also lead to typical hemiplegic gait [2]. Patients with post-stroke usually develop adaptive and compensatory locomotion strategies characterized by asymmetric performance and greater gait variability in spatiotemporal, kinematic, and kinetic parameters that require quantitative gait analysis systems to provide more accurate and robust measurements [4,5]. Throughout the rehabilitation process, periodical assessment of gait improvement in patients with stroke is crucial for clinicians to evaluate the effectiveness of treatment and to formulate subsequent treatment strategies [6].

Clinical gait analysis is often performed in a gait laboratory using three-dimensional motion capture systems based on infrared photoelectric cameras and reflective markers, which have been considered the gold standard for gait analysis [7]. However, laboratory gait analysis is restricted not only by space and cost but also by its dependence on skilled personnel to operate the devices [8]. In addition, during standard gait tests, in order to obtain more accurate data, subjects need to expose their skin for the accurate placement of markers, which can be a great inconvenience for some subjects [4].

With recent developments in sensing technology, more machines are now able to perceive, act, and interact with their environments, prompting it possible for depth-based perception technologies to provide a feasible solution for clinical gait analysis [9]. Microsoft Kinect V2, based on a depth camera, is the most representative example of this new technology and has been applied in the research of posture and motion capture. There have been numerous studies that support the reliability of the Kinect V2 in assessing spatiotemporal and kinematic gait parameters with good angle results and transverse displacement results for static posture testing, but the datasets used in these studies were relatively small [10,11,12,13]. However, Kinect obtains gait parameters through its processing software Kinect Software Development Kit (SDK), which is mainly aimed at researchers and developers and not very friendly to clinicians. The translation of Kinect into clinically practical and usable products is still in an early stage [14].

Odonate is a new intelligent gait analysis system developed by Maver Medical based on a depth camera for clinical application. It includes binocular color lenses, depth lenses, and depth learning systems. Different from traditional infrared three-dimensional (3D) motion capture systems, such as Vicon, the Odonate system does not require markers to be placed on the surface of the body or a professional test site, enabling rapid motion capture and gait analysis using a mobile terminal that occupies only one square meter. Therefore, Odonate can not only save time in clinical examination but also reduce the physical and mental pressure placed on patients during the testing procedure.

However, there are still some challenges before applying this system to clinical practice. To our knowledge, few previous studies have tested the validity and reliability of the Odonate gait analysis system by comparing it with traditional optics-based 3D gait analysis systems. In addition, individuals with motor dysfunction exhibit various gait patterns, and the question of whether Odonate can discriminate different gait patterns has yet to be answered.

Therefore, this study was designed to (1) evaluate the inter/intra-rater reliability of the Odonate gait analysis system for measuring spatiotemporal and kinematic parameters in both patients with post-stroke and healthy subjects, (2) to test the validity of Odonate compared with a reference 3D camera-based motion analysis system, and (3) further examine its ability to discriminate abnormal gait patterns.

## 2. Materials and Methods

### 2.1. Participants

A total of 16 patients with post-stroke (8 males; age: 40 ± 14 years; height: 172 ± 8 cm; weight: 72 ± 15 kg) recruited from the Beijing Rehabilitation Hospital, Capital Medical University, and 26 age-sex-matched healthy subjects free from lower limb disease (14 males; age: 42 ± 11 years; height: 171 ± 10 cm; weight: 72 ± 13 kg) were included in this study. The post-stroke participants were at least 6 months removed from a single major ischemic stroke (22 ± 8 months) and also capable of walking at least 60 m without assistance. Individuals who were unable to obey the instructions and patients with orthopedic or other neurological diseases leading to impaired walking ability were excluded from the study. All participants were informed of the study procedures and purposes and provided written informed consent. The study protocol was approved by the Ethics Committee of Beijing Rehabilitation Hospital, Capital Medical University (2021bkky-077).

### 2.2. Odonate Conditions

**Figure 1:**
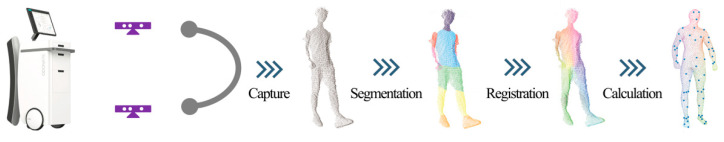
The analysis process of Odonate 3D motion capture system.

The Odonate 3D motion capture system, developed by Shanghai Maver Medical Technology Co., Inc. in China, comprises a mobile terminal (including two depth cameras with 30 Hz sample frequency) and a workstation (integrated with the depth sensing technology, depth neural network technology, and millimetric point cloud analysis technology). The system is equipped with a binocular depth camera combined with an artificial intelligence system to capture, analyze, and calculate gait parameters automatically. The analysis process is as follows: (1) capture, reconstructing the 3D movement model of participants based on depth perception technology; (2) segmentation, using deep learning technology to recognize human body segments automatically; (3) registration, automatically performing point cloud matching using deep neural networks; and (4) calculation, computing spatiotemporal and kinematic parameters (Figure 1).

### 2.3. Testing Procedures

**Figure 2:**
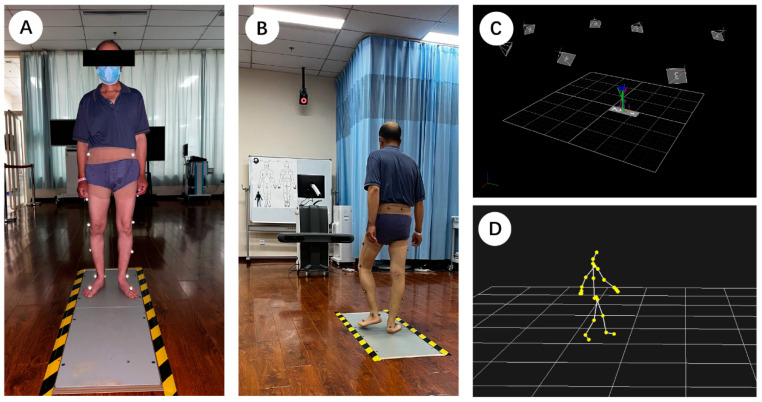
(**A**): Static calibration procedure of the Vicon system. (**B**): Simultaneous capture by Odonate and Vicon. (**C**): Data processing in Vicon Nexus software. (**D**): Data processing in Odonate software.

The tests in this study were conducted in a dedicated room with a clean environment that was free of distractions. Participants wore close-fitting, non-black, and non-reflective clothing to reduce capture errors. The testing consisted of three sessions. In the first session, two investigators (A and B) performed a standard gait test for all participants using the Odonate system to collect the data to be used for testing inter-rater reliability. Participants walked at their own comfortable speed until at least six successful trials were captured. The second session was started 24 h after the first. Investigator A performed the same test as in the first session using the Odonate system in order to provide data for intra-rater reliability. After participants rested for 10 min, the third session began. The Odonate system and an eight-camera 3D motion capture system (Vicon; Oxford, UK), with two embedded force platforms (AMTI; Watertown, MA, USA) were used simultaneously for synchronous measurement to examine the validity of Odonate. The frame of heel strike on the force platform was used to synchronize the measurements of the two systems. The sampling frequency of the Vicon system and force platform were 100 Hz and 1000 Hz, respectively. The plug-in gait model with 16 markers was used for the Vicon system to define body segments (Figure 2A,B).

### 2.4. Data Processing

Data measured by the Odonate system were processed by its analysis software to extract gait parameters, including the interception of gait cycle, marking of gait events, and calculation of gait parameters. The gait event detection on the videos recorded from each trial was made by manual frame-to-frame checking. The kinematic parameters calculated by the Odonate system were filtered by a 4th-order zero-lag 6 Hz Butterworth low-pass filter. The data collected by the Vicon system were processed in Nexus 2.10.1 software (Vicon; Oxford, UK), including the reconstruction of makers, gap filling, 6 Hz zero-lag low-pass filtering, marking of gait events, and calculation of gait parameters. Parameters common to the two systems were selected for subsequent analysis, including spatiotemporal parameters such as gait cycle time, step length, velocity, step cadence, stance phase, swing phase, and double support phase, and kinematic parameters such as angle curves of hip, knee, and ankle joints normalized to 100% of the gait cycle in the sagittal plane.

### 2.5. Statistical Analysis

The Pearson correlation coefficient, paired *t*-test and its effect size (Cohen’s d), Bland–Altman method consistency evaluation, and intra-group correlation coefficient (ICC) were used to determine the consistency and relationship strength of the measured spatiotemporal parameters in the Odonate system and between the Odonate and Vicon systems. Since high correlation between the measurement results of the two methods does not necessarily mean that the two methods are in good agreement, none of the above metrics alone should be employed as the sole statistic [15]. The Pearson correlation coefficient can reflect the correlation strength of the measurement results of the two methods, and the *t*-test and its effect size can detect the difference between the measurement results of the two methods. The Bland–Altman method and the ICC can measure the consistency of the two systems [16,17]. The Bland–Altman method has been used in many studies to judge whether there is absolute agreement between two systems. The narrower the 95% limits of agreement are, the more practical the use of the new system becomes [18]. The ICC is a useful quantitative indicator for describing the reliability and validity between two systems. In this study, the ICC of the type (2,1) was used to assess the agreement between the measurements of the Odonate and Vicon systems to validate the Odonate system for spatiotemporal parameters measurement [19]. The ICCs of the type (3,1) were also calculated to evaluate the inter/intra-observer reliability of the Odonate system for measuring spatiotemporal parameters. Values for the ICCs were interpreted as poor (<0.4), fair to good (0.4–0.74), or excellent (>0.75) [19].

The coefficient of multiple correlation (CMC) and a statistical parametric mapping (SPM) paired *t*-test were used to evaluate the inter/intra-observer reliability and validity of Odonate systems for measuring the kinematic curves of hip, knee, and ankle joint angles. The CMC can be used to calculate the similarity between different waveforms and takes into account the simultaneous influence of offset, correlation, and gain difference in its calculation process [20]. The CMC values were interpreted in the same way as the ICC. The CMC values could be explained as excellent similarity (0.95–1), very good similarity (0.85–0.94), good similarity (0.75–0.84), moderate similarity (0.6–0.74), and poor similarity (0–0.59)

The SPM method is a multi-dimensional data analysis technique that allows direct statistical analysis of one-dimensional waveform data. Its statistical outputs are represented as the original time series, providing an understanding of the periods during a gait cycle where significant differences may occur. In addition, the independent-sample SPM tests were used to compare the differences in kinematic curves between patients with post-stroke and healthy subjects measured by Odonate and Vicon, and the paired SPM tests were used to compare the differences between the affected and unaffected sides of patients with post-stroke [21].

The Odonate system’s ability to recognize abnormal gait was examined through the period where there were significant differences during a gait cycle identified by both Odonate and Vicon. The SPM analysis was performed using open-access SPM1D scripts (http://spm1d.org/ accessed on 1 October 2021; Pataky, 2012). All data processing and statistical analysis were performed using MATLAB software (MATLAB R2020b, Mathworks Inc., Natick, MA, US), and Python 3.8. Statistical significance was defined as *p* < 0.05.

## 3. Results

### 3.1. Gait Spatiotemporal Parameters

Since healthy subjects all walked symmetrically, gait parameters for each healthy subject were combined and averaged for their left and right sides. The spatiotemporal parameters of both healthy subjects and patients with post-stroke measured by the Odonate system showed excellent consistency for inter/intra-rater reliability (ICC_3,1_ = 0.801 to 0.988), though the *t*-test results showed statistical differences between different observers in the double support phases (*p* < 0.05, Table 1).

**Figure 3:**
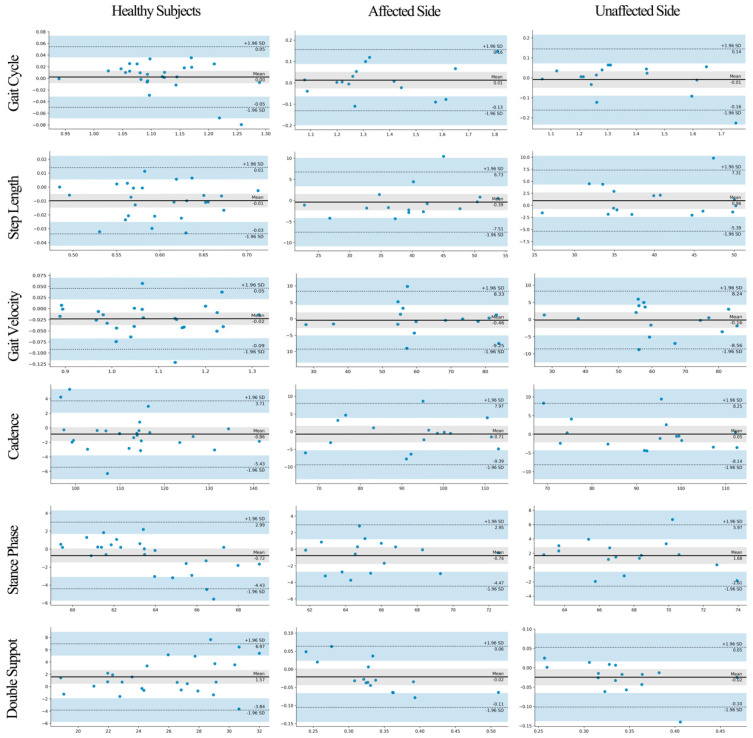
The Bland−Altman plots for the differences between the spatiotemporal parameters measured by Odonate and Vicon systems. The absolute differences between each data pair are plotted against their means. The two horizontal lines represent the 95% limits of agreement (range of error) and are calculated as 1.96 times the standard deviation of the mean difference between the two systems. The figure illustrates that the differences between the two systems lie evenly within the limits of agreement.

Compared to the Vicon system, the Odonate system showed statistical differences in the measurements of the double support phase for healthy subjects and of the stance phase, swing phase, and double support phase for the affected side for patients with post-stroke (*p* < 0.05), but the effect sizes of these differences were small (Cohen’s d = 0.395 to 0.496). Moreover, the measurement results of the two systems showed a significant correlation (r = 0.776 to 0.981) and consistency (ICC_2,1_ = 0.855 to 0.990), and the Bland–Altman results indicated that the differences between the two systems lay uniformly within the limits of agreement (Figure 3).

### 3.2. Joint Kinematics

**Figure 4:**
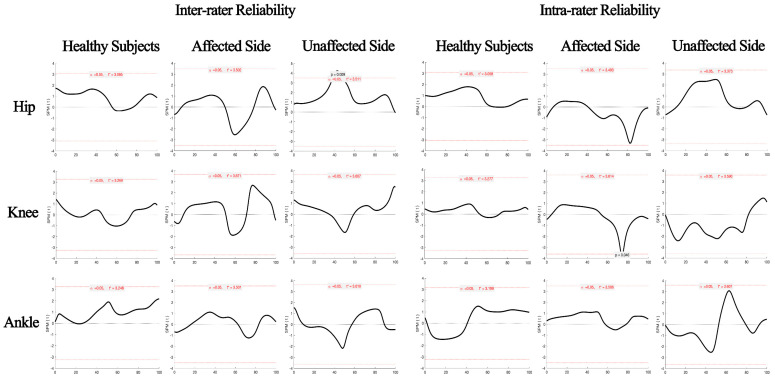
The SPM results of the inter/intra−rater reliability of the Odonate system for lower limb kinematics.

The SPM analysis results showed that there were no significant differences in hip, knee, or ankle joint angles between different observers and repeated measurements over almost the entire gait cycle (Figure 4). There was also excellent agreement between different observers and repeated measurements in hip, knee, and ankle joint kinematic curves (CMC = 0.909 to 0.999, Table 2).

**Figure 5:**
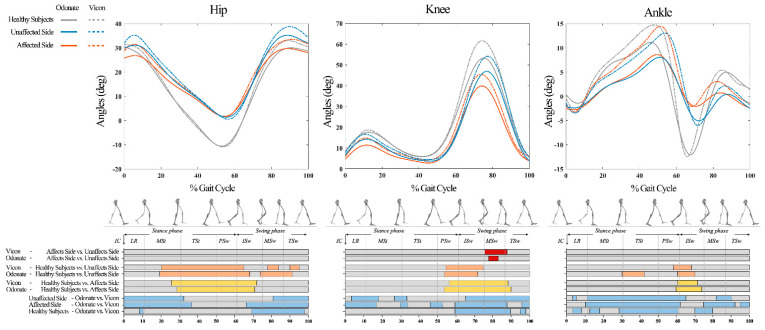
The lower limb kinematic curves measured by two systems for all participants. Significant differences between waveforms are indicated by the color bars under each graph. Blue color bars denote Odonate vs. Vicon on the Healthy Subjects, Affected Side, and Unaffected Side. Yellow, orange, and red color bars indicate the SPM independent−sample t−test results of each system for Healthy Subjects, Affected Side, and Unaffected Side, respectively.

Compared to the Vicon system, the Odonate system showed statistical differences during some stages of the gait cycle in the measurements of the hip, knee, and ankle joint angles for all healthy subjects, affected and unaffected sides of patients with post-stroke, exhibiting a smaller range of flexion angle, especially for the ankle joint (Figure 5). However, there was excellent agreement between the hip and knee joint kinematic curves measured by the two systems (CMC = 0.976 to 0.989, Table 2), but the agreement for the ankle joint kinematic curves was slightly lower (CMC = 0.868 to 0.917, Table 2). In addition, the stages with statistical differences between healthy subjects and the affected and unaffected sides of patients with post-stroke during a gait cycle also agreed between both systems (Figure 5).

## 4. Discussion

The present study tested the validity and inter/intra-rater reliability of the Odonate system for measuring gait spatiotemporal and kinematic parameters in healthy subjects and patients who had had ischemic strokes. Spatiotemporal parameters such as gait velocity, step length, cadence, and gait phase are important indicators to identify gait impairments caused by disease [1]. In this study, the repeatability tests of different raters and different days proved the excellent inter-rater reliability and intra-rater reliability of the Odonate system in measuring spatiotemporal parameters. The parameters of gait velocity, step length, cadence, and stride duration measured by the Odonate system showed extremely high consistency with the measurement by Vicon, indicating that these parameters measured by the Odonate system appear to be quite robust and could potentially be useful in clinical practice. However, the recognition capacity of the Odonate system was slightly lower in measuring some time-related parameters such as the stance phase, swing phase, and double support phase. These results were consistent with the findings from previous validation studies of similar gait measurement devices. Two literature review studies reported that the Kinect was exceptionally good for some spatial gait parameters such as step length, width, and asymmetry [22,23]; however, timing-related variables have not been shown to have such strong validity [24]. The main reason lies in the error of gait event detection, especially the error of toe-off events. Albert et al. [25] reported that the detection of toe-off events generally has larger errors compared to heel strike events in both Kinect v2 and its upgrades Azure Kinect. Xu et al. [26] also found that the temporal gait parameters based purely on heel strike have less error than that based on toe off. The reason for this result may be due to the positioning of the foot and camera, with the foot in front of the body when heel strike and the foot behind the body when toe off that could not be well observed because of blocking and camera perspective. Moreover, the Odonate system operated at a relatively low rate (30 Hz), which can result in large timing inaccuracy if errors of even a single frame are observed.

As we all know, kinematic curves are one-dimensional waveform data, so in order to facilitate statistical analysis, data analysis usually only focuses on specific events or abstract metrics that rely on parameters extracted from discrete points of gait waveform. Eltoukhy et al. [27] compared the agreement and consistency of kinematic parameters such as ankle, knee, and hip angle at the initial contact and the ROM, peak flexion, or extension angles during the stance and swing phase measured by Kinect v2 and BTS motion analysis system in patients with Parkinson’s disease. They found the consistency and agreement were excellent for all parameters of the knee and hip joints (ICC= 0.90 to 0.96) but poor for the ankle joint (ICC = 0.00 to 0.14). Tanaka et al. [28] compared the hip and knee joint angles at 11 discrete points (from 0% to 100% of the gait cycle in increments of 10%) throughout the gait cycle and concluded that the hip and knee joint measured by the Kinect v2 in the sagittal plane is acceptable, except for the knee joint angle from the first half of the stance phase. However, using predetermined parameters to verify undirected test hypotheses exposes the data to ‘regional focus bias’ [29,30,31]. Concentrating the analysis on discrete parameters, rather than the entire curve, may miss the differences along the time dimension and ignore the characteristics of gait data over time, resulting in increased Type I or Type II error (i.e., false positives or false negatives) [32]. In this study, the SPM method was used to compare the differences between joint angle curves, which has been proved to be able to avoid the above problems [31,32]. The repeatability tests of different raters and different days demonstrated the good inter-rater reliability and intra-rater reliability of the Odonate system in measuring kinematic curves.

When comparing the joint angles measured by the two systems, there were significant differences during some stages of the gait cycle, especially if the joint was at a higher angle of flexion, indicating that the Odonate system underestimated the flexion of the joint, and the greater the flexion angle, the greater the error. The reason for this result may be related to the different modeling methods of the two systems. Vicon used identified markers to calculate the centers of the joints, including an estimate of the center of the hip joint, and then established the rigid body coordinate systems to calculate the joint angle through rotation transformation. Odonate identified the 3D point cloud on the limb surface, extracting the surface key points, and solving the rotation transformation between the corresponding key points through the least-squares method to solve the joint angle. In addition, when the knee flexion angle is larger in the initial and mid-swing, the shank segment is located behind the body, and the angle between its long axis and the Odonate camera line-of-sight is smaller, which may increase the tracking error of the shank leading to greater knee angle measurement error. Nevertheless, the extremely high CMC indicated the tendency in the hip and knee joint angle over the gait cycle measured by the two systems was consistent.

Compared with the hip and knee joint angles, the ankle joint angle showed relatively poor validation results in this study, which is a widespread phenomenon in previous studies on the validation of the same type of devices [33,34]. Ma et al. [24] reported that the CMC values of hip and knee joint angle trajectories between Kinect and Motion Analysis were 0.81 and 0.87, respectively, after calibration, but even after calibration, the ankle flexion CMC value was only 0.43 (Table 3). Timmi et al. [35] found that the Kinect accurately captures hip and knee joint trajectories but not ankle joint trajectories. Eltoukhy et al. [27,33] also found similar results. One possible reason for the poor validation of ankle angle is that the long axis of the foot segment is short resulting in even a small tracking error may cause a large ankle angle error. Since the foot segment is located in the anterior–posterior direction of the body, unlike other body segments, tracking errors might arise due to the accompanying ground light reflection [24]. The close proximity of the foot segment, ankle joint, and the ground may also adversely affect the tracking of the ankle and foot [36]. Moreover, the smaller angle between the foot segment and the Odonate camera line-of-sight brings a great challenge to foot tracking, which makes the foot vision more easily blocked by the distal end and causes tracking errors. It has been reported that multi-camera fusion can alleviate these problems and improve tracking accuracy to a certain extent [22], which is also supported by this study. Since Odonate uses a binocular camera, the ankle joint angle recognition results in this study (CMC >0.87) were better than that of a single Kinect camera in the literature [24].

Whether a new gait device can identify abnormal gait patterns is an important aspect to verify the validity of its measurement. In this study, the SPM method was used to compare the joint angles of patients and healthy subjects measured by the two systems to confirm the ability of the Odonate system to recognize abnormal gait in patients with stroke. We found that the results identified by Odonate were basically consistent with that by Vicon in both hip, knee, and ankle joint angles, indicating that the ability of Odonate to recognize abnormal gait patterns in patients with stroke is comparable to that of Vicon, and it is an acceptable tool for clinical gait assessment.

Odonate has significant advantages that Vicon or other motion capture systems simply do not have. For Vicon and other systems, it takes thirty to sixty minutes to complete preparation before a test in addition to the time it takes to complete a formal test, and analysis, and to process and report gait results, and this time limits the use of motion capture systems in clinical practice. In contrast, Odonate takes less than five minutes to complete the whole process, benefiting from its intelligent capture and analysis process. In addition, Odonate could be used in a variety of situations, such as inpatient settings, clinics, and at home, not just in a laboratory. Compared with other depth camera-based devices of the same type, such as Kinect, which is mainly oriented to researchers and developers, Odonate is a system dedicated to clinical gait analysis. Its operation and interactive interface are more friendly to doctors, and it can directly give clinical reports.

However, Odonate still needs some upgrades. The recognition of timing-related parameters and the ankle joint angle need further study to improve its measurement accuracy. Odonate can only measure the sagittal joint angles, and the kinematics in the coronal and transverse planes should be further identified. Compared to Vicon, Odonate cannot calculate kinetic parameters. Although accessories are included with Odonate to capture and analyze plantar pressure, the hardware and associated algorithm should be redesigned so that it can achieve comparable results compared to the traditional force platform. The sampling frequency of the Odonate camera should be increased to improve its time resolution.

In addition to limitations with Odonate, there are several limitations in our study as well. Only patients with stroke and healthy subjects were included, and other populations should be enrolled in future investigations. Varieties of pathological gait patterns may present in different kinds of disease, not just stroke, and these other gait patterns could also be examined with Odonate. Furthermore, walking speed or tasks may also have affected gait patterns and caused variation, which should be further verified.

## 5. Conclusions

In conclusion, as a new gait analysis system, Odonate has excellent inter/intra-rater reliability and has strong validity in measuring some spatiotemporal parameters and the sagittal joint angles, except the gait phase division and the ankle joint angle. However, Odonate is comparable to the Vicon system in its ability to identify abnormal gait patterns in patients with post-stroke. Therefore, Odonate has the potential to provide accessible and objective measurements for clinical and out-of-laboratory gait assessment.

## Figures and Tables

**Table 1:** Mean, standard deviation, and the results of consistency analysis of spatiotemporal parameters.

Spatiotemporal Parameters		Odonate-Reliability			ICC_3,1_ (95%CI)		Odonate-Validity		Cohen’s d	r	ICC_2,1_ (95%CI)
		Day1-A	Day1-B	Day2-A	Inter-Rater	Intra-Rater	Odonate	Vicon			
healthy subjects											
	Gait Cycle (s)	1.111 ± 0.067	1.114 ± 0.079	1.114 ± 0.073	0.955 (0.899, 0.980)	0.968 (0.928, 0.986)	1.118 ± 0.070	1.115 ± 0.081	0.029	0.947	0.968 (0.929, 0.986)
	Step Length (m)	0.599 ± 0.057	0.602 ± 0.054	0.597 ± 0.051	0.974 (0.942, 0.988)	0.955 (0.899, 0.980)	0.594 ± 0.055	0.600 ± 0.055	0.167	0.956	0.977 (0.950, 0.990)
	Gait Velocity (m/s)	1.077 ± 0.117	1.081 ± 0.129	1.074 ± 0.111	0.985 (0.965, 0.993)	0.981 (0.958, 0.992)	1.069 ± 0.118	1.079 ± 0.112	0.186	0.974	0.987 (0.971, 0.994)
	Cadence (step/min)	113.481 ± 11.431	113.477 ± 11.662	112.951 ± 11.710	0.988 (0.974, 0.995)	0.988 (0.973, 0.995)	112.385 ± 11.196	113.245 ± 11.763	0.075	0.981	0.990 (0.977, 0.995)
	Stance Phase (%)	63.442 ± 2.546	62.726 ± 2.569	63.216 ± 2.260	0.942 (0.871, 0.974)	0.801 (0.555, 0.911)	63.262 ± 2.196	63.979 ± 3.304	0.256	0.838	0.872 (0.714, 0.943)
	Swing Phase (%)	36.558 ± 2.546	37.274 ± 2.569	36.784 ± 2.260	0.942 (0.871, 0.974)	0.801 (0.555, 0.911)	36.738 ± 2.196	36.020 ± 3.304	0.256	0.838	0.872 (0.714, 0.943)
	Double Support (%)	26.911 ± 5.073	**25.431 ± 4.669 ^a^**	26.573 ± 4.742	0.931 (0.845, 0.969)	0.872 (0.714, 0.943)	26.525 ± 4.374	**24.960 ± 3.516 ^b^**	0.395	0.776	0.863 (0.694, 0.938)
Affected Side											
	Gait Cycle (s)	1.346 ± 0.204	1.350 ± 0.191	1.365 ± 0.213	0.983 (0.953, 0.994)	0.932 (0.805, 0.976)	1.367 ± 0.219	1.354 ± 0.205	0.059	0.942	0.969 (0.912, 0.989)
	Step Length (m)	0.401 ± 0.088	0.404 ± 0.076	0.400 ± 0.085	0.973 (0.923, 0.991)	0.980 (0.944, 0.993)	0.400 ± 0.092	0.403 ± 0.081	0.045	0.920	0.954 (0.869, 0.984)
	Gait Velocity (m/s)	0.622 ± 0.160	0.6112 ± 0.160	0.616 ± 0.166	0.983 (0.951, 0.994)	0.984 (0.954, 0.994)	0.614 ± 0.159	0.618 ± 0.163	0.028	0.962	0.980 (0.944, 0.993)
	Cadence (step/min)	93.393 ± 14.693	92.837 ± 13.937	92.126 ± 14.455	0.984 (0.955, 0.995)	0.958 (0.881, 0.985)	92.122 ± 14.493	92.832± 14.333	0.049	0.953	0.976 (0.931, 0.992)
	Stance Phase (%)	65.614± 2.290	64.932 ± 2.484	65.689 ± 3.248	0.855 (0.585, 0.949)	0.837 (0.533, 0.943)	65.176 ± 2.861	65.936 ± 2.869	0.265	0.782	0.877 (0.649, 0.957)
	Swing Phase (%)	34.386 ± 2.288	35.068 ± 2.484	34.311 ± 3.248	0.855 (0.585, 0.949)	0.837 (0.533, 0.943)	34.824 ± 2.861	34.064 ± 2.869	0.265	0.782	0.877 (0.649, 0.957)
	Double Support (%)	35.062 ± 5.499	**33.063 ± 6.304 ^a^**	36.465 ± 5.994	0.889 (0.681, 0.961)	0.832 (0.518, 0.941)	32.689 ± 4.995	34.806 ± 7.912	0.320	0.873	0.881 (0.660, 0.959)
Unaffected Side											
	Gait Cycle (s)	1.350 ± 0.204	1.367 ± 0.216	1.346 ± 0.188	0.967 (0.905, 0.988)	0.971 (0.916, 0.990)	1.355 ± 0.188	1.363 ± 0.219	0.043	0.938	0.962 (0.891, 0.987)
	Step Length (m)	0.398 ± 0.092	0.396 ± 0.084	0.387 ± 0.071	0.979 (0.938, 0.992)	0.840 (0.543, 0.944)	0.403 ± 0.077	0.393 ± 0.075	0.126	0.910	0.953 (0.865, 0.984)
	Gait Velocity (m/s)	0.613 ± 0.161	0.609 ± 0.160	0.627 ± 0.163	0.980 (0.943, 0.993)	0.987 (0.962, 0.995)	0.618 ± 0.156	0.620 ± 0.163	0.010	0.965	0.982 (0.947, 0.994)
	Cadence (step/min)	93.123 ± 14.365	92.041 ± 14.486	92.991 ± 13.540	0.982 (0.948, 0.994)	0.984 (0.954, 0.994)	92.386 ± 13.160	92.332 ± 14.559	0.004	0.960	0.977 (0.934, 0.992)
	Stance Phase (%)	68.054 ± 3.737	68.344 ± 3.914	67.452 ± 3.598	0.946 (0.846, 0.981)	0.887 (0.678, 0.961)	68.521 ± 3.196	**66.842 ± 3.562 ^b^**	0.496	0.795	0.883 (0.665, 0.959)
	Swing Phase (%)	31.946 ± 3.737	31.656 ± 3.914	32.548 ± 3.598	0.946 (0.846, 0.981)	0.887 (0.678, 0.961)	34.479 ± 3.196	**33.158 ± 3.562 ^b^**	0.496	0.795	0.883 (0.665, 0.959)
	Double Support (%)	35.113 ± 5.815	**32.543 ± 5.532 ^a^**	33.328 ± 5.532	0.905 (0.729, 0.967)	0.837 (0.532, 0.943)	32.820 ± 4.521	**35.257 ± 6.384 ^b^**	0.441	0.792	0.855 (0.586, 0.949)

Note: ^a^ indicates Day1-A and Day1-B were significantly different (*p* < 0.05). ^b^ indicates Odonate and Vicon were significantly different (*p* < 0.05), and Pearson correlation coefficients were all significantly different.

**Table 2:** CMC results of the Odonate system in lower limb kinematics.

Variable	Inter-Rater Reliability	Intra-Rater Reliability	Validity
**Healthy Subjects**			
Hip	0.999	0.996	0.989
Knee	0.999	0.995	0.977
Ankle	0.983	0.977	0.917
**Affected Side**			
Hip	0.982	0.987	0.977
Knee	0.953	0.963	0.978
Ankle	0.946	0.909	0.868
**Unaffected Side**			
Hip	0.989	0.985	0.988
Knee	0.982	0.980	0.976
Ankle	0.934	0.950	0.917

**Table 3:** The validation of some gait analysis systems based on depth camera for measuring kinematics compared with conventional optical 3D gait analysis systems in the literature.

Authors	Systems	Subjects	Conditions	Consistency	Kinematics	Values
Ma et al. [24]	Kinect v2 VS. Motion Analysis	10 children with cerebral palsy	Walk	CMC	Hip flexion/extension	0.75 to 0.81
					Knee flexion/extension	0.85 to 0.87
					Ankle dorsi/plantarflexion	0 to 0.43
Eltoukhy et al. [27]	Kinect v2 VS. BTS System	11 healthy subjects and 8 patients with Parkinson’s Disease	Walk	ICC (Consistency and Agreement)	Hip ROM	0.86 to 0.98
					Knee ROM	0.69 to 0.98
					Ankle ROM	0.13 to 0.28
Eltoukhy et al. [33]	Kinect v2 VS. BTS System	10 healthy subjects	Walk with different speeds	ICC (Consistency and Agreement)	Hip ROM	0.77 to 0.86
					Knee ROM	0.68 to 0.82
					Ankle ROM	–0.39 to 0.05
Oh et al. [34]	Kinect v2 VS. BTS System	12 healthy subjects	Stair ascent and descent	ICC (Consistency and Agreement)	Peak hip angle	0.86 to 0.97
					Peak knee angle	0.54 to 0.95
					Peak ankle angle	–0.26 to 0.33
Timmi et al. [35]	Kinect v2 VS. Vicon	20 healthy subjects	Fast walk	Range of LOA	Hip marker coordinates (x,y,z)	(7.7, 10, 8.3) mm
					Knee marker coordinates (x,y,z)	(8.7, 12.3, 11.6) mm
					Ankle marker coordinates (x,y,z)	(10.8, 15.1, 26.2) mm

## Data Availability

The raw data supporting the conclusions of this article will be made available from the corresponding author on reasonable request.
